# Growth-oriented thinking and career decision-making self-efficacy among university students: the chain mediation role of mindfulness and future time perspective

**DOI:** 10.3389/fpsyg.2025.1599906

**Published:** 2025-12-02

**Authors:** Qian Li, Xiang Wang, Xuzhou Li, Keli Yin

**Affiliations:** Faculty of Education, Yunnan Normal University, Kunming, China

**Keywords:** career decision-making self-efficacy, growth-oriented thinking, mindfulness, future time perspective, university students

## Abstract

**Introduction:**

To explore the relationship between growth mindset and career decision-making self-efficacy among university students as well as the underlying influencing mechanisms.

**Methods:**

It uses the Growth Thinking Scale, Mindfulness Scale, Future Time Perspective Scale, and Career Decision Self Efficacy Scale to conduct a survey of 670 university students.

**Results:**

The results show: A significant positive correlation between self-efficacy and growth-oriented thinking in career decision-making (*r* = 0.468, *p* < 0.01), mindfulness (*r* = 0.298, *p* < 0.01), and future time perspective (*r* = 0.352, *p* < 0.01). The mediating pathways of growth-oriented thinking influence these factors are statistically significant (0.015, 0.042, and 0.076, respectively), as are their chain relationships. In this model, the mediating effect of future time perspective on growth-oriented thinking and self-efficacy in career decision-making is greater than that of mindfulness.

**Conclusion:**

The results indicate that mindfulness and future time perspective can explain the impact of university students’ growth-oriented thinking on their career decision-making self-efficacy.

## Introduction

The website of the Ministry of Education of China has announced that the number of university students in 2022 exceeded ten million. As the number of students in the country continues to rise, finding employment is becoming an increasing problem, which affects individual well-being and social harmony. University students are at an important turning point in their lives, and need to make important decisions about their careers and employment (Kulcsár et al., 2020). At this stage, they explore, choose, and commit to a profession as they enter society. Career decision-making self-efficacy is an individual’s belief in their ability to make effective career-based decisions, and directly affects the career choices of students (Taylor and Betz, 1983). Some researchers state that career decision-making self-efficacy is the self-evaluation or confidence of decision-makers in the necessary abilities to successfully complete various tasks during the career decision-making process (Betz et al., 1996; Hampton, 2006). Studying career decision-making self-efficacy can help mitigate the sense of hopelessness students face as they make decisions about the future. It has important theoretical significance and practical value, as it enriches the literature and improves the effectiveness of decision-making.

Growth mindset is a concept proposed by American psychologist Dweck based on attribution theory, achievement goal theory, and implicit intelligence theory. Different from fixed thinking, she believes that growth thinking is a belief that one’s own attributes such as intelligence and ability can be continuously developed through individual efforts (Dweck, 2006a). Individuals with high levels of growth mindset have higher levels of life satisfaction and job satisfaction (Lam and Zhou, 2020; Van Tongeren and Burnette, 2016;), as well as stronger self-efficacy (Yu et al., 2022). This means that shaping growth mindset can help individuals cultivate self-efficacy, as individuals with higher growth mindset have stronger motivation to pursue success. Growth mindset plays an important role in individuals’ sustained efforts to achieve their commitment to goals (Lai et al., 2022). Therefore, according to existing research, growth mindset is an important predictor of career decision-making self-efficacy.

According to the theoretical framework of Cognitive-Behavioral Therapy, thinking influences individual behavior through beliefs and cognition, and growth-oriented thinking can promote positive behavior in individuals. Previous studies suggest that mindfulness, which can enhance self-efficacy, may be an important mediating factor (Lu et al., 2021). Mindfulness refers to an individual’s conscious and uncritical attention to internal and external stimuli at a given moment, including their physical sensations and emotional reactions (Bishop et al., 2004; Purser and Milillo, 2014; Lau et al., 2006). Self-determination indicates that humans have a positive instinct and innate potential for self-actualization and continuous integration (Ryan and Deci, 2002). This suggests that people are more likely to achieve work-related tasks if these tasks correspond to their own values and interests (Cagney and Lauderdale, 2002). Although the relationship between growth-oriented thinking and mindfulness is yet to be directly confirmed, it can be inferred that the “ability growth view” it stimulates may encourage self-awareness, and allow individuals to increase mindfulness by consciously focusing on their feelings, emotions, and thinking. Some research has shown that mindfulness is positively correlated with self-efficacy, and can promote its improvement (Svartdal et al., 2021; Da Silva et al., 2020). The study of mindfulness among kindergarten teachers found that mindfulness could positively predict self-efficacy, but believed the relationship between mindfulness and career decision-making self-efficacy needed further testing (Cheng et al., 2022). The present study therefore proposes Hypothesis 1: Growth-oriented thinking has an impact on career decision-making self-efficacy through the mediating effect of mindfulness.

Further, future time perspective (FTP) may play a role in the relationship between growth-oriented thinking and career decision-making self-efficacy. FTP refers to the cognitive, emotional, and behavioral tendencies exhibited by individuals when anticipating, planning, and constructing possibilities for future social and self-development (Kooij et al., 2016; Wang et al., 2021; Li et al., 2023). Some research found that individuals could enhance their FTP by developing their growth-oriented thinking. Individuals with growth-oriented thinking believe that their abilities can be shaped and cultivated; they tend to set future-oriented goals, thereby indicating a higher level of FTP (Zhao et al., 2021). According to the Goal Setting Theory, established goals drive individual behavior, which also drives FTP (Locke, 1968; Schunk, 1989). It is therefore possible for the latter to play a role in the relationship between growth-oriented thinking and career decision-making self-efficacy. Because of its influence on the individual acquisition of knowledge and information, FTP can also have a direct effect on career decision-making self-efficacy (Liang et al., 2017). This study therefore proposes Hypothesis 2: Growth-oriented thinking has an impact on career decision-making self-efficacy through the mediating effect of FTP.

This study explores the mediating role of mindfulness and FTP in growth-oriented thinking and career decision-making self-efficacy, and explores the correlation between the former two. Mindfulness can stimulate an individual’s future hopes by improving FTP, and Wittmann et al. (2014) found that individuals with developed mindfulness skills had better FTP. Other studies have shown that individuals with high levels of mindfulness are more likely to establish a sense of time, which promotes their ability to utilize and manage it, thereby enhancing their FTP. These studies show that a high level of mindfulness predicts a developed FTP, which could help university students make effective decisions to achieve their future goals (Su et al., 2021). This study therefore proposes Hypothesis 3: mindfulness and FTP have a chain mediating effect on career decision-making self-efficacy.

**Figure 1:**
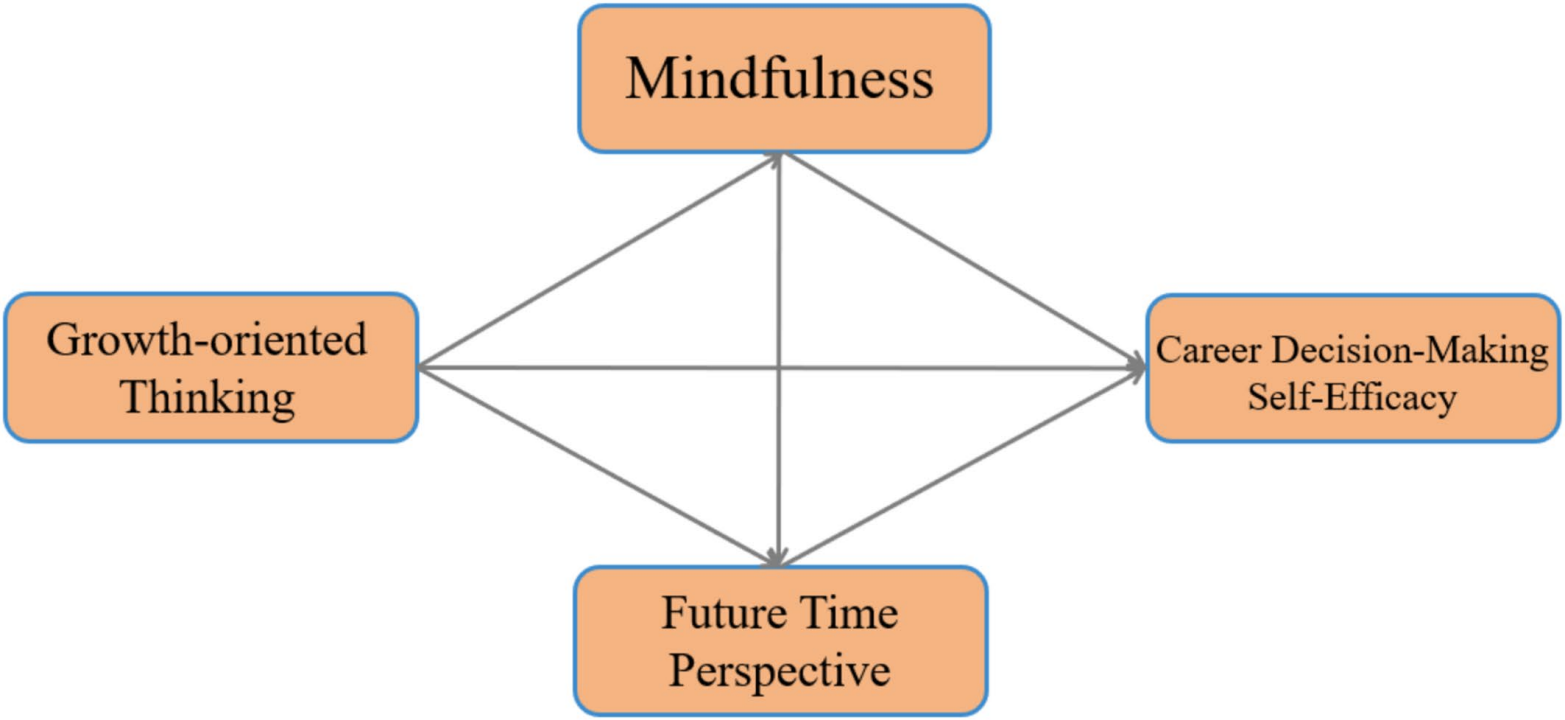
Hypothetical model of the mediation chain of mindfulness and FTP.

There is currently a lack of empirical evidence to prove the relationship between growth-oriented thinking and career decision-making self-efficacy, and the mechanism between the two. This study addresses the shortfall by constructing a multiple mediation model for growth-oriented thinking, mindfulness, FTP, and career decision-making self-efficacy, which can provide a basis for targeted intervention strategies for career decision-making self-efficacy in the future. The theoretical hypothesis model of this study is shown in Figure 1.

## Participants and methods

### Participants

The sample size was determined by using G*Power software, a medium effect size of 0.15, *α* of 0.05, and statistical power of 0.95. The minimum sample used in this study was 472 respondents. This study conducted a survey of 700 university graduates from four universities using a convenient sampling method and an online questionnaire. After excluding invalid answers such as incomplete or randomly answered questionnaires, a total of 670 valid questionnaires were obtained, with an effective rate of 96%. Among these, 238 respondents were male (35.52%) and 432 were female (64.47%); 295 students from liberal arts majors (44.03%), 204 students from science and engineering majors (30.45%), 118 students from art majors (17.61%), and 53 students from medical majors (7.91%). The details are shown in Table 1. The average age and standard deviation of respondents were 22.06 ± 1.23 (M ± SD). The participants in this study were all voluntary and obtained informed consent. The study complied with all ethical and legal requirements, prioritizing the safety and rights of the participants. The research project has obtained ethical approval for the degree program in Applied Psychology from the Education Department of Yunnan Normal University. The questionnaires were completed anonymously, and no personal information such as names or student ID numbers was collected.

**Table 1:** Demographic characteristics of the participants.

Demographic variables	Category	*N*	Percentage
Gender	Male	238	35.52%
	Female	432	64.47%
Grade	Freshman	509	75.97%
	Sophomore	89	13.28%
	Junior	59	8.81%
	Senior	7	1.04%
	Master’s students	5	0.75%
	Doctoral students	1	0.15%
Professional	Liberal arts	295	44.03%
	Science and engineering,	204	30.45%
	Arts	118	17.61%
	Medicine	53	7.91%

### Tools

#### Growth mindset scale (GMS)

The Growth Mindset Scale (GMS) was developed by Dweck (2006b) which translated in Chinese vision and applied by Jia (2018). It uses a Likert 4-point scoring system for a total of 20 questions, with questions 1, 4, 7, 8, 11, 12, 14, 16, 17, and 20 being scored in reverse. In the study, the critical ratio method was used to conduct item analysis on the items of the scale, in order to obtain the decision value CR value of individual items. Items 7, 8, and 14 that did not reach a significant level after item analysis were deleted. A score of 1 indicates a strong bias toward fixed thinking; 2 indicates a somewhat bias toward fixed thinking; 3 indicates a somewhat bias toward growth-oriented thinking; and 4 indicates a strong bias toward growth-oriented thinking. The Cronbach *α* was 0.61, indicating the scale has good reliability.

#### Mindful attention awareness scale (MAAS)

Brown and Ryan (2003) developed the Mindfulness Attention Awareness Scale. The Chinese vision of the scale was revised by Chen et al. (2012), which comprises 15 items. Likert 6-point scoring was used, with a high score reflecting increased awareness of and attention to the present in daily life (Liu et al., 2023). The *α* value of 0.94 indicates good reliability.

#### Zimbardo time perspective inventory (ZTPI)

The future subscale of the Zimbardo Time Perspective Inventory (ZTPI) was used to measure individuals’ cognition, planning, and tendencies toward the future (Zimbardo and Boyd, 1999). And the Chinese vision of it was revised by Wang (2016). This subscale uses Likert 5-point scoring, with 1 indicating “very inconsistent” to 5 indicating “very consistent.” The scale has a total of 5 items. The higher the score, the higher the level of future time perspective. In this study, the scale’s *α* value of 0.74 indicates good reliability.

#### Career decision-making self-efficacy scale (CDMSE)

This study adopted the Career Decision Self-Efficacy Scale for College Students, developed by Peng and Long (2001). The scale has five dimensions – self-evaluation; information collection; goal selection; planning; and problem solving – and uses Likert 5-point scoring. This study’s α value was 0.99, indicating good reliability.

### Data analysis

Data analysis in this study was conducted using SPSS 27.0, MPLUS 8.3, and Hayes’ PROCESS macro 4.0. First, the Kaiser-Meyer-Olkin (KMO) test and Bartlett’s test of sphericity were performed to assess the suitability of the data for factor analysis. All assumptions were checked: Multicollinearity (VIF < 2.5), Residual normality (Shapiro–Wilk *p* > 0.05), Bootstrapping (5,000 samples) for indirect effects. The KMO value was 0.974, and Bartlett’s test was significant (*p* < 0.05), indicating that the data were appropriate for subsequent factor analysis (Chiou et al., 2022). Next, Harman’s one-way test was used to check for common methodological biases and the results showed no serious bias. Descriptive analyses were then conducted to summarize participants’ demographic characteristics. Cronbach’s alpha coefficients were calculated using SPSS and all of these coefficients were located between 0.60 and 0.90, indicating reliability of the scales. To verify structural validity, a Confirmatory Factor Analysis (CFA) was performed, and the model showed good fit, with indices such as *x*^2^/*df*, CFI, TLI, NFI, IFI, RMSEA, and SRMR all meeting conventional standards. Detailed results of the reliability and validity tests are presented in Table 1. Pearson correlation analysis was conducted to assess associations between variables. Finally, mediation effects were tested using Hayes’ PROCESS macro, employing bootstrapping with 5,000 samples to calculate 95% confidence intervals (CI) (Shrout and Bolger, 2002). A mediation effect was considered significant if the 95% CI did not include zero.

## Results

### Common method deviation testing

The self-reported evaluation method used in the survey of variables in this study may have led to a common method bias. To mitigate this, the Harman single factor test method was used to conduct a common method bias test (Tang and Wen, 2020). Exploratory factor analysis was conducted on all variables, and the first principal component explained the variance variation to a degree of 39.10 percent, which was below the critical value of 40 percent. This shows that there was no significant issue of common method bias in this study.

### Analysis of difference in career decision-making self-efficacy across different demographic variables

The differences in career decision self-efficacy across various demographic variables were analyzed. An independent samples t-test was conducted for gender, and an analysis of variance (ANOVA) was performed for grade level and major. As shown in Table 2, gender (*t* = 3.124, *p* < 0.01) indicated that males had significantly higher levels of career decision-making self-efficacy than females (134.43 > 126.54), while grade (*F* = 1.691, *p* > 0.05) and major (*F* = 1.366, *p* > 0.05) did not show significant differences.

**Table 2:** Analysis of difference in career decision-making efficacy

**Variables**	**Category**	***M±*SD**	***F/t***
Gender	Male	134.43±32.55	3.124**
	Female	126.54±28.90	
Grade	Freshman	129.64±31.01	1.691
	Sophomore	122.96±28.16	
	Junior	137.04±29.53	
	Senior	125.64±22.51	
	Master’s students	131.00±16.17	
	Doctoral students	106.5	
Professional	Liberal arts	127.25±28.50	1.366
	science and engineering,	130.32±32.12	
	Arts	133.62±32.40	
	Medicine	127.34±29.67	

*p < 0.05, **p < 0.01, ***p < 0.001.

### Correlation analysis of variables

Pearson correlation analysis was used to examine the relationships among gender, Growth mindset, MAAS, ZTPI, and Career decision self-efficacy. As shown in Table 3, all key study variables were positively correlated. Moreover, gender displays a significant negative correlation with the other variables.

**Table 3:** Correlation analysis results for variables and their dimensions.

Variables	*M*	*SD*	1	2	3	4	5
1. Gender	1.64	0.479	1				
2. Growth-oriented thinking	45.32	4.172	−0.120**	1			
3. Mindfulness	66.27	12.699	−0.144**	0.298**	1		
4. Future time perspective	17.19	3.305	−0.121**	0.352**	0.295**	1	
5. Career decision-making Self-Efficacy	129.34	30.460	−0.124**	0.468**	0.329**	0.429**	1

GMS, growth mindset; MAAS, mindful attention awareness scale; ZTPL, zimbardo time perspective inventory; CDMSE, Career decision-making self-efficacy.

*p < 0.05, **p < 0.01, ***p < 0.001.

### The mediating effects analysis

Previous studies have shown that gender can influence college students’ career decision-making self-efficacy (Liu et al., 2023). Therefore, gender level was included as control variables to test the mediating roles of mindful attention awareness and future time perspective in the relationship between growth mindset and career decision-making self-efficacy among college students. Model 6 of the SPSS PROCESS macro 3.5 by Hayes was used, with 5,000 bootstrap samples and 95% confidence intervals (CI) computed. The regression results are presented in Table 4: growth mindset significantly positively predicted career decision-making self-efficacy (*β* = 0.327, *p* < 0.001), mindful attention awareness (*β* = 0.285, *p* < 0.001), and future time perspective (*β* = 0.285, *p* < 0.001). mindful attention awareness significantly positively predicted future time perspective (*β* = 0.202, *p* < 0.001). When growth mindset, mindful attention awareness, and future time perspective were entered simultaneously to predict career decision-making self-efficacy, both mindful attention awareness (*β* = 0.148, *p* < 0.001) and future time perspective (*β* = 0.266, *p* < 0.001) showed significant positive effects on career decision-making self-efficacy. At this point, the positive predictive effect of growth mindset on career decision-making self-efficacy remained significant (*β* = 0.327, *p* < 0.001), indicating that the chain mediating roles of mindful attention awareness and future time perspective were supported. The results are shown in Table 4.

**Table 4:** Results of intermediary model regression analysis.

Outcome variable	Predictor variable	*R*	*R2*	*F*	*β*	*t*
CDMSE	GMS	0.473	0.224	96.268	0.460	13.390***
	gender				−0.069	−2.009*
MAAS	GMS	0.317	0.101	37.366	0.285	7.709***
	gender				−0.110	−2.961**
ZTPL	GMS	0.408	0.167	44.397	0.285	7.651***
	MAAS				0.202	5.421***
	gender				−0.057	−1.601
DMSE	GMS	0.565	0.320	78.137	0.327	9.317***
	MAAS				0.148	4.298***
	ZTPL				0.266	7.598***
	gender				−0.032	−0.974

GMS, growth mindset; MAAS, mindful attention awareness scale; ZTPL, zimbardo time perspective inventory; CDMSE, Career decision-making self-efficacy.

*p < 0.05, **p < 0.01, ***p < 0.001.

**Figure 2:**
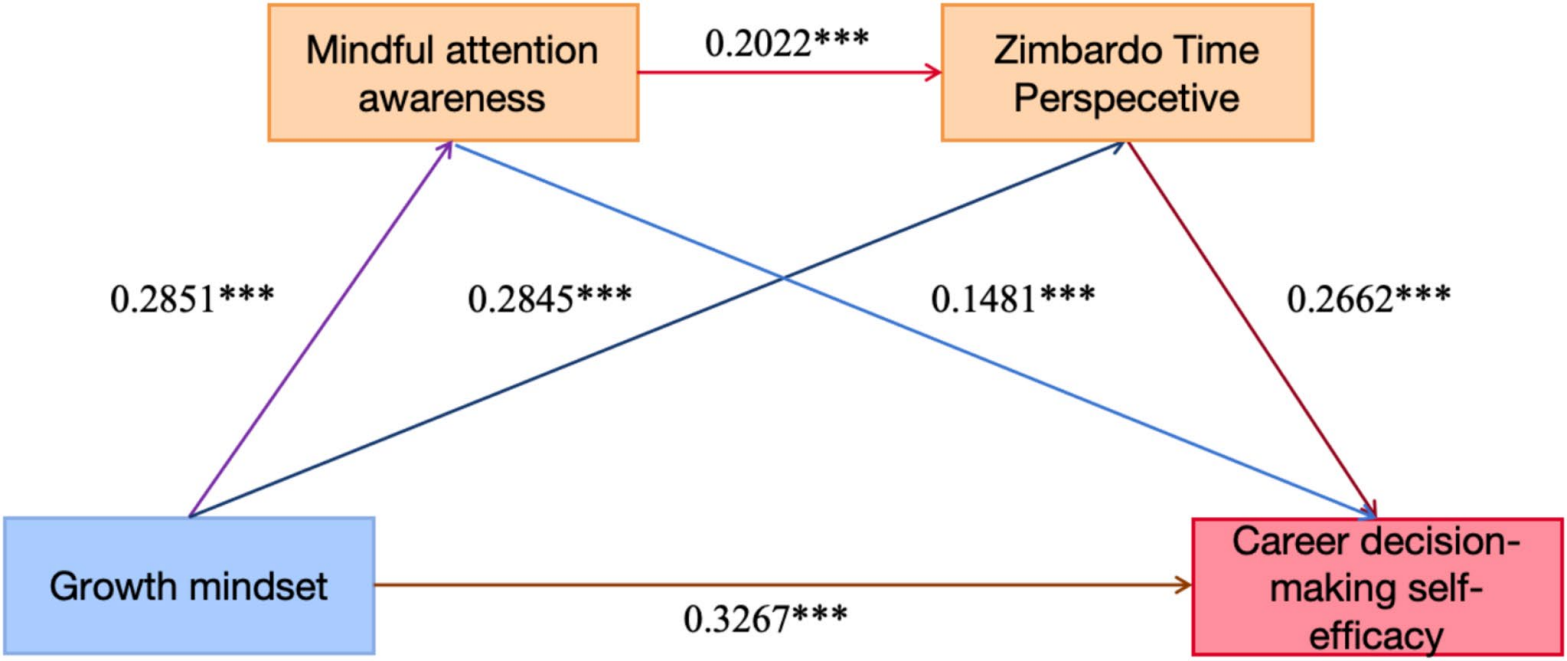
Regression and chain mediation models (*p < 0.05, **p < 0.01, ***p < 0.001).

Further mediation analysis results are shown in Table 5 and Figure 2. The direct effect of growth mindset on career decision-making self-efficacy was 0.327, with the confidence interval does not contain zero, indicating a significant direct effect and accounting for 71.02% of the total effect. The total indirect effect was 0.133, also with a confidence interval, indicating that the mediating effects of mindful attention awareness and career decision-making self-efficacy were significant, accounting for 28.98% of the total effect. Specifically, the indirect path through mindful attention awareness alone had an effect size of 0.042, 95% CI = [0.017, 0.073]; through future time perspective alone, the effect size was 0.076, 95% CI = [0.042, 0.115]; and through the chain mediation of mindful attention awareness and future time perspective, the effect size was 0.015, 95% CI = [0.006, 0.028]. These results indicate that all three indirect paths were statistically significant.

**Table 5:** Mediation effect test results.

Model effect	Effect size	B Boot SE	95% CI Effect		Effect proportion
			LLCI	ULCI	
Total effect	0.460	0.034	0.393	0.528	
Direct effect: GMS →DMSE	0.327	0.035	0.258	0.396	71.02%
Total indirect effect	0.133	0.021	0.093	0.177	28.98%
GMS → MAAS → CDMSE	0.042	0.014	0.017	0.073	9.17%
GMS → ZTPL → CDMSE	0.076	0.019	0.042	0.114	16.46%
GMS → MAAS → ZTPL → CDMSE	0.015	0.006	0.006	0.028	3.33%

GMS, growth mindset; MAAS, mindful attention awareness scale; ZTPL, zimbardo time perspective inventory; CDMSE, Career decision-making self-efficacy.

## Discussion

### Gender differences in self-efficacy in career decisions

The research findings indicate that there are significant gender differences in career decision-making self-efficacy. Specifically, men typically exhibit higher career decision-making self-efficacy than women. This finding aligns with previous studies, which have shown that male college students generally possess higher self-efficacy (Yu et al., 2022). According to Hofstede’s cultural dimensions theory, masculine cultures emphasize achievement and competition, which may reinforce men’s self-efficacy, while women may experience reduced self-assessment due to cultural pressures (Hofstede, 2011). Additionally, traditional Chinese cultural norms of “men working outside the home and women managing the household” may also influence these differences. However, such disparities are not biologically determined but rather stem from modifiable environmental factors. This suggests that future research should adopt longitudinal designs to track the impact of cultural changes and implement targeted interventions to promote gender equality in career development.

### The impact of growth-oriented thinking on career decision-making self- efficacy among recent students

The results showed a significant positive correlation between growth mindset and career decision-making self-efficacy among college students. Even after accounting for the mediating variables, the positive predictive effect of growth mindset on career decision-making self-efficacy remained significant, indicating that as the level of perceived growth mindset increased, the level of career decision-making self-efficacy also significantly rose, thus supporting Hypothesis 1. This finding is consistent with prior research. and indicates that students with higher levels of growth-oriented thinking have stronger career decision-making self-efficacy (Yu et al., 2022; Qian, 2025). growth mindset influences students’ career decision-making self-efficacy through multiple mechanisms. First, Dweck’s mindset theory organizes goals, attribution, helplessness, and effort belief into a meaningful system (Dweck, 2019), which can illustrate the predictive relationship between growth-oriented thinking and career decision-making self-efficacy. Because some scholars view growth-oriented thinking as a series of attributes such as intelligence, ability, and personality, they believe it that can be changed through effort. According to this theoretical explanation, students with high levels of growth-oriented thinking have more self-belief when making decisions that affect their careers.

Additionally, based on self-efficacy theory, a growth mindset directly influences self-efficacy by reframing individuals’ interpretations of career setbacks. When individuals view career challenges as opportunities for growth, they can activate one of the sources of self-efficacy, namely performance accomplishments (Bandura, 1977).

In terms of teaching and curriculum systems, educators should provide developmental feedback, design progressive challenges tailored to students’ current ability levels, clearly identify areas for improvement in career counseling, and point out directions for breakthroughs in the next stage. The curriculum system should incorporate a progressive career competency ladder, from basic industry knowledge to job skills training to decision-making and risk assessment, with clear competency standards set for each stage to ensure that students gradually improve their belief in their effectiveness through controllable challenges.

### The mediating role of mindfulness between growth-oriented thinking and career decision-making self-efficacy

The research found that mindfulness played a mediating role between growth-oriented thinking and career decision-making self-efficacy among university students; i.e., a higher level of the former led to increased skills in the latter. This confirms previous research that mindfulness can benefit individuals by enhancing psychological function (Shi et al., 2023). The ability of mindfulness to reduce avoidance behavior and redundant and negative thinking give students effective psychological energy for career decision-making; promote positive behavior; and reduce difficulties (Aydoğmuş, 2022). Conversely, students with low levels of mindfulness may face greater difficulties in career decision-making, which can affect their career choices and employment (Arslan, 2020). Mindfulness therefore plays an important mediating role between growth-oriented thinking and career decision-making self-efficacy among university students, and suggests that psychological health educators should actively observe mindfulness levels and experiences among students. Previous studies have shown that group mindfulness training can enhance individual self-efficacy (Su and Luo, 2024). In learning and daily life, psychological health education methods such as group training can be used to enhance the mindfulness level of university students, thereby enhancing their career decision-making self-efficacy when they graduate. Future research can be conducted based on the I-Sustainability Design Thinking (ISDT) concept (Chiou et al., 2021) to design group counseling or individual interventions aimed at cultivating and enhancing college students’ mindfulness and spiritual well-being (Liu et al., 2021).

### The mediating role of FTP between growth-oriented thinking and career decision-making self-efficacy

The study found that FTP played a mediating role between growth-oriented thinking and career decision-making self-efficacy among university students; i.e., a higher level of growth-oriented thinking enhanced the former, thereby improving the latter. This confirms Hypothesis 2. High levels of growth-oriented thinking predict stronger FTP, of which planning, striving for, and achieving goals are characteristics (Zimbardo and Boyd, 1999). FTP is therefore an important condition for improving career decision-making self-efficacy, Gao and Dai found that it could enhance students’ professional decision-making self-efficacy by improving their time management skills (Gao and Dai, 2019). It can be seen that the higher an individual’s level of growth-oriented thinking, the better their FTP, which assists their self-efficacy in career decision-making. This means that university students’ growth-oriented thinking can indirectly affect their career decision-making self-efficacy by influencing their FTP. According to Social Emotional Choice theory, individuals with higher levels of FTP have advantages in terms of expanding their social circle and enriching social network practices, which can bring more potential employment opportunities (Liang et al., 2017). This indicates that individuals with strong FTP are more willing to strive, believe in, and pursue their career goals.

### The chain mediation role of mindfulness and FTP

This study found that mindfulness and FTP played a chain mediating role between growth-oriented thinking and career decision-making self-efficacy among university students; i.e., high levels of growth-oriented thinking enhance mindfulness, thereby enhancing FTP and increasing career decision-making self-efficacy. This supports Hypothesis 3. Career decision-making is a source of stress among students, and according to Folkman et al.’s Psychological Stress and Coping Theory, the cognition of individuals influences their coping behavior. Previous studies have shown that mindfulness has a positive impact on individuals’ ability to perceive the future (Nuño and Shelton, 2022; Chiesa and Serretti, 2009), which indicates a positive relationship between mindfulness and FTP. The present study also found that mindfulness and FTP played a mediating role between growth-oriented thinking and career decision-making self-efficacy, indicating that they are important affecting factors. Finally, the mediating effect of FTP was found to be more pronounced than that of mindfulness. Mindfulness purposefully regulates an individual’s current cognition, which is a consciousness generated by the uncritical presentation of each moment’s experience in the present moment (Kabat-Zinn, 2002); FTP is a bridge that connects the present and future, and individuals’ current expectations of future goals confirm its importance in enhancing career decision-making self-efficacy. Efforts should therefore be made to cultivate university students’ growth-oriented thinking; improve their FTP through mindfulness; guide them to have a positive attitude and confidence in the future; and motivate them to pursue their goals persistently. This also provides educators with some methods to better guide graduates, graduates can enhance career efficacy by: mindfulness training to reduce decision anxiety, FTP workshops to clarify long-term goals, growth mindset interventions to embrace challenges. Incorporate future planning modules (such as career planning workshops and future scenario simulations) into college or vocational training programs to help students practice setting long-term goals and developing actionable pathways to achieve them.

## Conclusion

(1) Growth mindset can positively predict career decision-making self-efficacy; (2) Mindfulness positively predicts future time perspective; (3) Mindfulness and future time perspective play a chain mediating role between growth thinking and career decision self-efficacy. This study confirms that a growth mindset enhances college students’ self-efficacy in career decision-making through a chain of mediating effects involving mindfulness and future time perspective. This mechanism aligns with Bandura’s self-efficacy theory, which posits that cognitive restructuring and the accumulation of stage-based achievements jointly reinforce decision-making confidence.

### Research limitations and recommendations

While this study elucidates the formation mechanism of college students’ career decision-making confidence through the sequential mediation of growth mindset, mindfulness, and future time perspective, several limitations warrant attention. Methodologically, the reliance on cross-sectional design precludes causal inferences among variables. Implementing longitudinal research designs would enable more robust examination of the dynamic relationship between developmental cognitive patterns and subsequent job-search behaviors, thus providing stronger evidence for establishing causal mechanisms.

The sampling strategy presents two constraints: geographical concentration (limited to institutions within one region) and institutional homogeneity (lacking diversity in university tiers). To enhance the representativeness of findings, future studies should employ stratified sampling across three institutional tiers: vocational colleges, provincial universities, and elite institutions. This multi-level approach would significantly improve the generalizability of research recommendations.

Compared to previous studies, our research findings confirm and complement the existing research in this field. Partial discrepancy may stem from our focus on undergraduates (vs. graduates), whose career concerns are more urgent. Future research should examine developmental stage effects.

Multiple factors influence university students’ career decisions. Future studies could expand the research scope by incorporating additional variables that impact career decision-making self-efficacy. External support systems—including familial assistance (manifested through digital career consultations) and institutional provisions (such as university career services’ interview training programs)—demonstrate significant interaction effects with psychological preparedness in career decision-making processes. We propose an integrated research framework examining how psychological capital (confidence, adaptability) and environmental enablers (mentorship, skill-building) synergistically facilitate career transitions—akin to how both competence and proper tools determine task performance. The career decision-making of college students is a vertical development process that covers the entire academic career during their university education and continues to extend into their career after graduation. It is recommended that higher education institutions integrate mindfulness training and goal-setting tools into career education programs to guide students in transforming career challenges into opportunities for skill development.

Other research has found that mindfulness, spiritual well-being, and subjective well-being are positively correlated, with spiritual well-being mediating the relationship between mindfulness and SWB (Liu et al., 2020). Therefore, future interventions could further explore the impact of mindfulness on the psychological states of graduating college students. Loving-kindness meditation has been shown to effectively enhance SWB and spiritual well-being, but its underlying mechanisms—including its effects on mindfulness, spiritual well-being, SWB, and other psychological variables—require further elucidation.

## Data Availability

The data that support the findings of this study are available from the corresponding author upon reasonable request. Requests to access the datasets should be directed to Qian Li, liqian@ynnu.edu.cn.
